# Release Retardation of Model Protein on Polyelectrolyte-Coated PLGA Nano- and Microparticles

**DOI:** 10.1371/journal.pone.0092393

**Published:** 2014-03-19

**Authors:** Chandra Nugraha, Meghali Bora, Subbu S. Venkatraman

**Affiliations:** School of Materials Science & Engineering, Nanyang Technological University, Singapore, Singapore; National University of Ireland, Galway, Ireland

## Abstract

PEM capsules have been proposed for vehicles of drug microencapsulation, with the release triggered by pH, salt, magnetic field, or light. When built on another carrier encapsulating drugs, such as nanoparticles, it could provide additional release barrier to the releasing drug, providing further control to drug release. Although liposomes have received considerable attention with PEM coating for sustained drug release, similar results employing PEM built on poly(lactic-co-lycolic acid) (PLGA) particles is scant. In this work, we demonstrate that the build-up pH and polyelectrolyte pairs of PEM affect the release retardation of BSA from PLGA particles. PAH/PSS pair, the most commonly used polyelectrolyte pair, was used in comparison with PLL/DES. In addition, we also demonstrate that the release retardation effect of PEM-coated PLGA particles diminishes as the particle size increases. We attribute this to the diminishing relative thickness of the PEM coating with respect to the size of the particle as the particle size increases, reducing the diffusional resistance of the PEM.

## Introduction

It is well known that polyelectrolyte multilayer (PEM) film built directly on drug crystals to form capsules could serve as a diffusion barrier to the encapsulated drug [1], [2]._ENREF_1 Since its inception in 1998 [3], PEM capsules have been proposed for vehicles of drug microencapsulation, with the release triggered by pH, salt, magnetic field, or light [4].

When built on another carrier encapsulating drugs, such as nanoparticles, it could provide additional release barrier to the releasing drug, providing further control to drug release. Of these particles, liposomes have received considerable attention with PEM coating for sustained drug release [5]–[10]. With size ranging from ∼100–500 μm, these PEM-coated liposomes have been reported to exhibit more sustained drug release compared to its naked counterpart. Doxorubicin, an anti-cancer agent, for instance, was reported to have reduced release from 60% to 35% at 12 h with poly(allylamine hydrochloride) (PAH)/poly(acrylic acid) (PAA) coating [6], while release of pyranine, a hydrophilic fluorescent dye, is almost completely diminished with poly-l-Lysine (PLL)/Poly(l-aspartic acid) (PASA) coating [8]. Release of BSA has also been retarded in the order of days, employing chitosan (CHI)/alginate (ALG) coating [10].

Similar results employing PEM built on poly(lactic-co-glycolic acid) (PLGA) particles for sustained release, however, have been scant. Although PLGA has been used commonly as a biodegradable drug delivery matrix, with wide-ranging application in genetic materials [11], protein [12], and anti-cancer drug delivery [13], most employ PEM on PLGA particles for surface functionalization [14], [15], improved transfection capability [16], or cellular uptake studies [17]–[19], leaving release control as a side note. While some kinetic-related works on PEM-coated PLGA exist, they are few and far in-between.

Zhou *et al*, for instance, reported PEM coating that either increases or decreases the rate of release of carboxyfluorescein slightly on PLGA nanoparticles depending on the charge of the outer layer [20]. Our own group has previously reported progressive release retardation of fluorescein isothiocyanate (FITC)-dextran with increasing number of layer of PEM on PLGA nanoparticles in timescale of days [21].

The study on release control and therefore, permeability of coatings built on PLGA particles, is still lacking. Herein, we investigate the correlation between the build-up parameters of PEM when built on PLGA particles and release retardation, since it is known that build-up parameters such as pH and polyelectrolyte types affect the morphology of PEM, employing PAH/Poly(sodium 4-styrenesulfonate) (PSS) as commonly used polyelectrolyte pair and PLL/dextran sulfate (DES). We also investigate the effect of PLGA particle size on the release retardation.

PLL and DES have been employed for PEM assembly with each other or other polyelectrolytes for cell-related applications, such as guiding cell differentiation [22], [23], changing cell behavior [24], or creating enzymatically degradable microcapsules [25]. The suitability of PLL/DES in this study extends to the fact that just as PAH/PSS, they are a weak and strong polycation and polyanion combination, respectively. The polycation has a similar pKa values to PAH (∼9) [26], [27], while the polyanion is strongly ionized throughout the pH region of interest here (pKa < 2) [28], similar to PSS.

## Materials and Methods

### Materials

PLGA 50/50, indicating a lactic/glycolic acid composition of 50%/50% (mol), consists of two different IV: 1.18 dL g^−1^ from Bioinvigor and 1.01 dL g^−1^ from Purac.

Two types of poly(vinyl alcohol) (PVA) were used to create the particles: PVA-403 was purchased from Kuraray, with a hydrolysis rate of 78% − 82% and MW ∼ 15 000 Da according to the manufacturer’s specification, and PVA 30000 was purchased from Sigma-Aldrich, with a hydrolysis rate of 87% − 90% and MW of 30000 − 70000 Da.

Bovine serum albumin (BSA) and the polyelectrolytes used: PAH (MW ∼ 56000 Da), PSS (MW ∼ 70000 Da), PLL hydrobromide (MW 30000 − 70000 Da), and DES (MW 36000 − 50000 Da), were purchased from Sigma-Aldrich. HPLC-grade dichloromethane (DCM) and dimethyl sulfoxide (DMSO) were purchased from Tedia, with Millipore water being used for all the experiments. All salts for buffer preparations—phosphate buffer saline (PBS) and carbonate buffer—were of analytical grade, purchased from Sigma, except NaCl and KCl, from VWR, and Na_2_HPO_4_, from Alfa Aesar.

### Particle fabrication

Particle fabrication follows the double-emulsion method. In general, BSA was dissolved in PBS and emulsified into PLGA solution. Probe sonication for 2 min was always employed for all the particles to form the primary emulsion, with or without 0.5% Coumarin 6. This emulsion was further emulsified into an external PVA solution, the method of which depends on the formulation. The organic solvent was subsequently evaporated with or without additional PVA for particle hardening. The solidified particles were then subject to removal of residual PVA by repeated centrifugation, and were immediately used for subsequent procedures or freeze-dried. The full list of the fabrication parameters for each type of particle is presented in Table 1. The calculated percentage in the table was always based on weight/volume.

**Table 1:** Particle fabrication parameters.

Type of particles	BSA	PLGA	PVA	Emulsion II	Evaporation
Nanoparticles	0.4 mL, 3%	IV 1.18 dL g^−1^, 2.5%	10 mL, 1% PVA 30000	2 min sonication	Overnight, 1000 rpm, 20 mm bar
60 or 160 μm particles	0.2 mL, 12%	IV 1.01 dL g^−1^, 5% or 10%	50 mL, 0.3% PVA-403 + 5% NaCl	2 h, 900 rpm, 20 mm bar	3 h, final PVA totaling 2 L
2 μm particles	0.2 mL, 12%	IV 1.01 dL g−1, 5%	60 mL, 0.3% PVA-403 + 5% NaCl	2 h, 900 rpm, 40 mm bar	Overnight, final PVA totaling 80 mL

### BSA quantitation

BSA was quantitated using the Micro BCA protein assay kit from Thermo Scientific with subsequent absorbance detection at 562 nm. Both fluorescence spectroscopy and absorbance detection for BSA were done on Infinite M200 (Tecan). In all measurements the calibration curves were made with the corresponding solvents.

### In-vitro drug release

Particles were dispersed into microtubes containing 1 mL of PBS (pH 7.4, and in some cases with 0.01% Tween 20) or carbonate buffer (pH 9.6), and incubated at 37 °C with a horizontal orbital shaker shaking at 50 rpm. At specific time points particles were separated from the supernatant by centrifugation, and aliquots of the supernatant were taken for quantitation specific to each model drug. The same amount of buffer was replenished to its original volume. The amount quantitated was calculated for cumulative amount of model drug released from the particles.

The content of model drugs in the particles was liberated by digestion through dispersion in 1 M of NaOH and incubation at 37°C overnight. Once digested, quantitation of the model drug content was done according to each drug’s quantitation protocol. An additional step of pH adjustment to ∼7 was done prior to quantitation. The drug loading then is defined as follows. 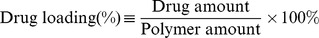



### PEM build-up on particles

Polyelectrolytes were dissolved in 0.5 M NaCl with a concentration of 1 mg mL^−1^. For microparticles, the particles were suspended in the polyelectrolyte solution for 5 min and washed with either water or 0.5 M NaCl twice before the next layer build-up of the oppositely charged polyelectrolyte.

For nanoparticles, particles were re-dispersed in 0.5 mL of water first before addition of 0.5 mL of polyelectrolyte solution due to difficulty in re-dispersion in high ionic strength solution. The coating time was adjusted to 5 min per layer. The pH of the polyelectrolyte solution is indicated whenever adjusted. The washing in-between each coating for nanoparticles was done once in pH-adjusted/un-adjusted water. For particles re-dispersed with sonication, sonication was done for 10 min in an ice bath to prevent temperature increase.

### PEM build-up confirmation

For particles, layer build-up was monitored after every one layer prior to a single washing with water. Laser Doppler Micro-electrophoresis for *ζ*-potential measurements were done with the Malvern Zetasizer Nano ZS (Malvern Instruments) at 25°C. Measurement of *ζ*-potential was done in folded capillary cells with water as dispersant, calculated using the Henry equation with Smoluchowski approximation. The sign reversal of the *ζ*-potential value indicates PEM build-up.

### Microscopy

Particle and film morphology was characterized with JSM-6360 and JSM-6340F SEM (both from JEOL). Microparticles and films were coated with gold or platinum before mounting on carbon tape before imaging. The size of micron-sized particles was measured by imaging through SEM and analysis on ImageJ with a minimum of 500 particles using area-equivalent diameter. Confocal laser microscopy (CLSM) (Leica LAS) was used to visualize the extent of aggregation in PBS on particles loaded with Coumarin 6. Particles for visualization purpose was freeze-dried in 1% sucrose solution to protect against aggregation. This concentration had been shown to retain the size of particles. The particles were washed in DI water repeatedly before dispersion in PBS.

DLS for z-average hydrodynamic radius and PDI measurements were done on Zetasizer Nano ZS (Malvern Instruments). The size distribution was calculated using a built-in auto-correlation function with a backscatter detector at 173°. Size progression during coating of nanoparticles was measured in PBS for correlation with the respective release curves, unless otherwise indicated. For microparticles, the dispersant was water.

### Statistical analysis

Release studies, loading, size, and *ζ*-potential measurements were done in triplicate. Statistical analysis to compare significant difference between curves were done with GraphPad Prism using two-way ANOVA with repeated measures. To compare two values, Student’s t-test was done. Significant difference is confirmed at *p*<0.05.

## Results and Discussion

### Particle fabrication and LBL coating

**Figure 1:**
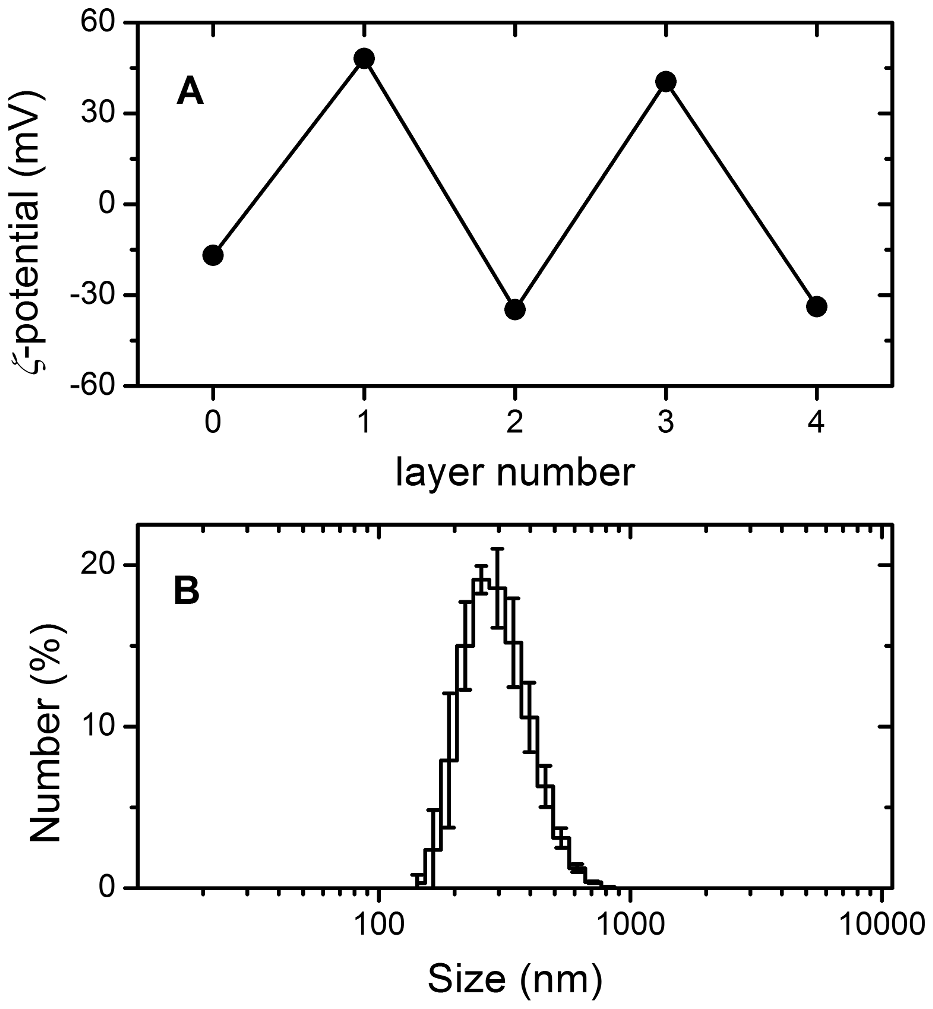
Typical particle size distribution and *ζ*-potential evolution of nanoparticles. Alternating signs on the ***ζ*** -potential confirms PEM build-up (A). The particle size distribution is of particles prior to any coating (B). Those coated show a shifted distribution.

BSA-loaded nanoparticle formation was confirmed through size measurement with a *z*-average of 330 nm. The size ranges from 100 nm to less than 1 μm (Figure 1B). The particles exhibit an encapsulation efficiency of 68%, and a *ζ*-potential of −25 mV. The *ζ*-potential of particles coated with PLL/DES showed considerably smaller value than those coated with PAH/PSS, because of the lower charge density. For comparison, the absolute value on PAH/PSS could reach as high as 40 mV, while it is only 15 mV on PLL/DES.

Nanoparticle coating with polyelectrolyte has been reported to experience aggregation after several layers. For instance, with only 2 layers consisting of PAA/PAH, it was reported that liposome size increased from 250 nm–520 nm [6]. On smaller liposomes, the particles increased from an original size of 110 nm to 140 nm with PAH coating and to 230 nm with a final PAA coating [9]. After several layers, the size stops increasing [29], [30]. When monitored with zetasizer, our increment in nanoparticle size resulted in higher PDI. To create a more reflective evaluation of the state of aggregation, therefore, nanoparticles after coating of each layer were monitored with CLSM instead.

Particles for CLSM visualization were fabricated as the counterpart of nanoparticles used for the drug loss and release studies, with the exception of Coumarin 6 incorporation for contrast agent for confocal microscopy. Due to the low amount of loading (0.5% theoretical loading), it is expected that it has little effect on the aggregation behaviour of the coated particles.

**Figure 2:**
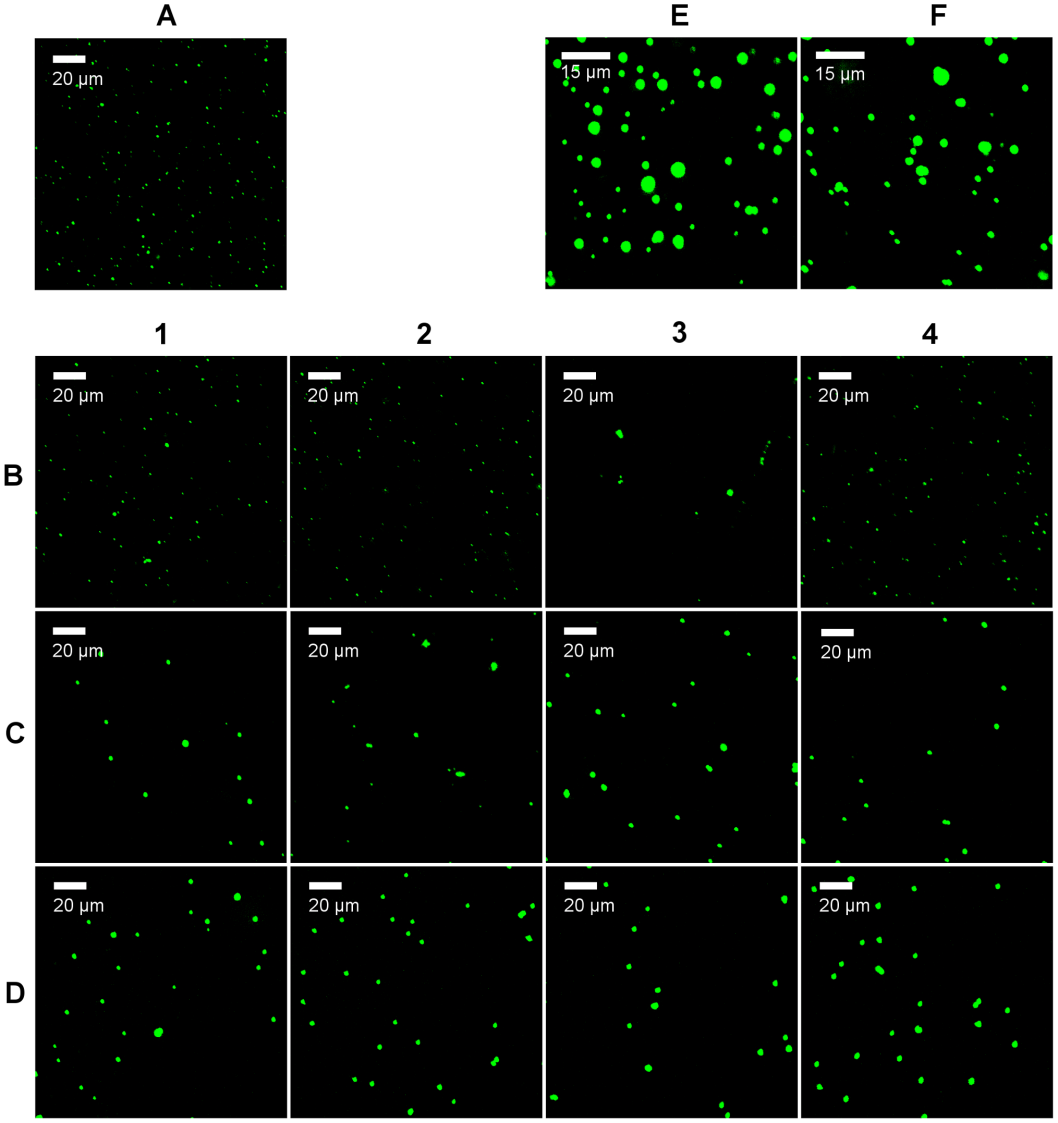
Extent of particle aggregation in PBS. CLSM images of original uncoated nanoparticles (A), coated nanoparticles with PLL/DES at pH 4 (B1-B4), with PLL/DES at pH 9 (C1-C4), and with PAH/PSS without pH adjustment (D1-D4). The numbers indicate the number of layers. Larger, uncoated particles at ∼2 μm (D) with their coated counterparts at 4 layers (E) are without aggregation.

The visualization was done at low concentration to avoid aggregation artifacts. Particles at higher size were also fabricated to determine the size dependence of the release retardation. Figure 2A shows the uncoated nanoparticles. PLL/DES coating at pH 4 (Figures 2B) does not result in aggregation except at layer 3; whereas PLL/DES at pH 9 (Figures 2C) and PAH/PSS (Figures 2D) result in an aggregated state that is visible from the CLSM.

The increase of aggregation on PLL/DES at pH 9 compared to pH 4 is caused by a nearer pH to p*K_a_*. It has been reported that closer inspection on the morphology of PEM coated on polystyrene nanoparticles revealed larger thickness and rougher morphology as the p*K_a_* value is approached, resulting in high aggregation [30]. To minimize aggregation, therefore, the particle size was increased (Figure 2E), and upon coating with PAH/PSS at 4 layers, no such aggregation was found.

But although aggregation exists in microparticles and nanoparticles, the former, which are in the order of 60 μm and above, consists of PLGA fusion above its *T_g_* at 37 °C. It does not occur spontaneously, rather, only after a few days, and does not affect coated particles due to barrier of chain diffusion by PEM. On nanoparticles, in contrast, the aggregation is due to the charge of the respective particle, the interaction between the polyelectrolyte chain on one particle and another, and occurs instantaneously after dispersion in the respective medium.

As a side note, aggregation due to polyelectrolyte coating derives from a different origin from uncoated particles. The latter is governed by the classical DLVO theory, whereas the former is in the first place due to patches of uneven polyelectrolyte ‘decoration’ on the surface, creating regions with non-uniform sign of charge [31], [32]. This creates electrostatically attractive forces between patches of surfaces that are dissimilar in charge, and is the origin of aggregation in polyelectrolye-coated particles. It is known, for example, that gold nanoparticles [33], latex particles [34], and liposomes [29] aggregate in the presence of polyelectrolyte.

Although this phenomenon has been investigated extensively in a situation where aggregation occurs during incremental polyelectrolyte addition into nanoparticle suspension, and is dictated by the *ζ*-potential—having maximum aggregation near neutral charge—an increased, irreversible aggregation could occur due to maximum proximity between particles effected by centrifugation. Even without polyelectrolyte treatment, PLGA nanoparticles became aggregated with repeated centrifugation, with an increase of size from 330 nm to 400 nm after 2 rounds of centrifugation.

### Drug loss during coating

It is known in some literature that release is reduced after certain treatment with additional coating. Unfortunately, without quantification of the amount of drug lost during such treatment, it is probable, that a reduced drug release is the result of drug loss, instead of the additional coating. For illustration, consider the following scenario. In a 100 μg-drug-containing particle, drug amount released after some duration is 20 μg, registered as 20%. Suppose that due to drug loss, the release is now reduced to 5 μg due to 15 μg dug loss. The drug content now is 85 μg, and the release is now registered as 5.9%. At the outset, 5.9% of the 20% drug released within the same period of time could appear as a reduction of release caused by release retardation of the coating, simply because this had been calculated based on the measured drug content. This understanding, however, eludes the truth of the matter. The reduced drug release is in fact caused by drug loss, even when the release profile is calculated based off the drug content. This argument still also stands even if the resulted release is > 15 μg, *i.e*., release should still appear lower percentagewise.

For this matter, BSA amount lost during coating was quantified using calibration standards in its own respective solutions. PAH especially presents another potential complication due to insoluble complex formation with BSA. This occurs, however, at comparable BSA/PAH ratio, and reduces significantly when either component is added or removed from the solution [35].

Visible complex formation also forms when in mixture with BCA reagents, possibly due to the change in ionic strength, but disappears upon incubation at 37°C. The calibration curve however, although significantly higher than in PBS at low concentration (<37.5 μg mL^−1^), still retains its linearity, and converges with that in PBS at high concentration. Variations due to pH were also anticipated, with pH showing no significant difference on the calibration curve at the range found in the coating processes. PAH concentration during coating was assumed to remain constant due to excess, after taking into account an approximate Γ value of 0.3 mg m^−2^ [36].

**Figure 3:**
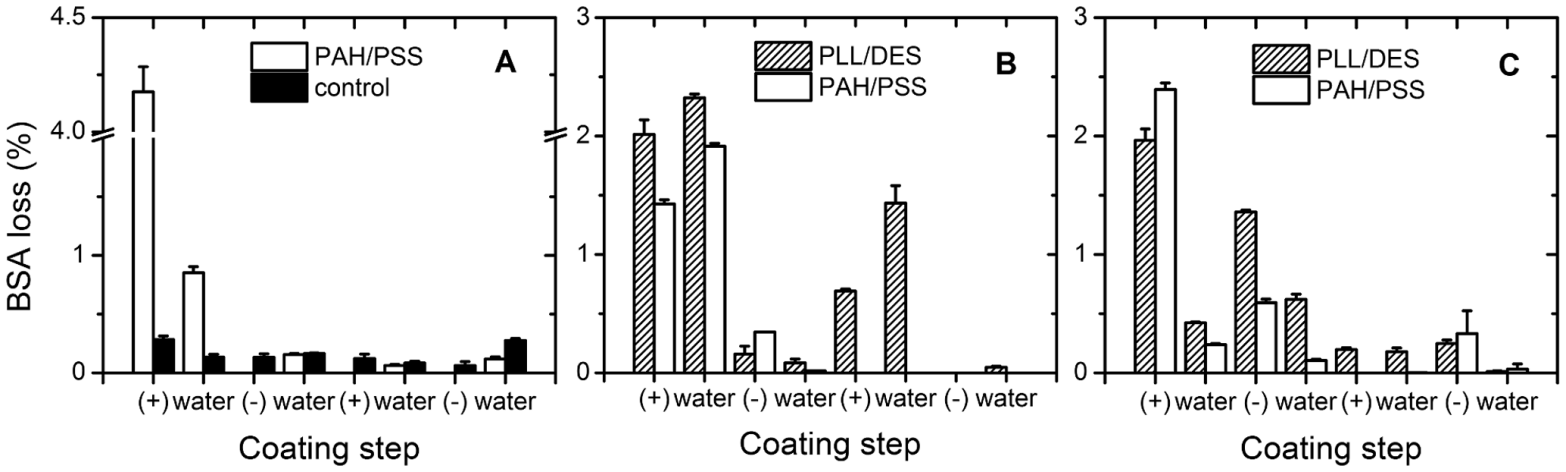
BSA amount lost during coating. BSA amount was quantified during build-up of PEM at unadjusted pH with PAH/PSS (A), PLL/DES at pH 4 (B), and PLL/DES at pH 9 (C). As a control, particles underwent the same routine of repeated washings and centrifugations, but with water used throughout the routine instead of polyelectrolyte solutions.

Due to its much increased surface area, BSA quantification becomes more significant during the coating process, and the reduction in release could be caused by a simple reduction of drug amount. For this reason coating process was monitored for drug loss amount. It was expected that the amount of BSA lost could be exorbitant. The amount however, is maximum across our different samples only at 8% loss for PLL/DES coating. For other coating conditions, the values are provided in Table 2, with the lowest being PAH/PSS coating at pH 4 and 9, at 3.7%. The amount of BSA lost is also greatest at the first few steps, and decreases with increasing layer number (Figure 3). Attempts to reduce loss by pre-coating it during fabrication with PAH in the outer PVA solution, for instance, did not succeed because of minimal BSA encapsulation as a result. In any case, the total drug lost during coating of all the samples average to a value of 5.4%.

**Table 2:** Total BSA loss amount.

	Polyelectrolyte coating	
Build-up pH	PAH/PSS (%)	PLL/DES (%)
NA	8.0±0.4	5.4±0.1
4	6.8±0.2	3.7±0.0
9	5.0±0.1	3.7±0.2

NA: Not adjusted. Values are calculated based on drug content.

The amount lost during coating is significantly higher than the control (Figure 3A). Although FITC-dextran control was done in PBS and not water, thereby rendering disagreement in data not fully comparable, it is suspected that BSA, being charged, desorbs from the particle surface more readily by electrostatic interaction with polyelectrolyte solution, whereas the same does not occur in water.

Furthermore, depending on the coating pH, BSA loss during the first polyelectrolyte adsorption could be either higher or lower than during its washing step. At pH 9, BSA amount lost during the first PAH adsorption is 2.4%. The subsequent washing step has the value drop to 0.2%. At pH 4, in contrast, the amount increases from 1.4% to 1.9%. The same trend is also found during PLL/DES coating, where BSA lost decreases during subsequent washing step only at pH 9, but not 4. This could be attributed to the p*I* of BSA, ∼4.7 [37], below which it acquires a positive charge. At pH 9 when BSA is negative, it could be more easily bound to the previously adsorbed positive PAH or PLL, hence the lack of BSA detected during the subsequent wash. Conversely, at pH 4, the reverse occurs.

The amount lost in PLL/DES is also greater than in PAH/PSS at any pH. It appears at the outset that the higher the lost amount, the higher the reduction, presuming the reduction in release is caused by a reduction of BSA amount in the particle. This however, is not true, as shown by correlation of drug release and drug loss on particles coated with PAH/PSS and PLL/DES.

**Figure 4:**
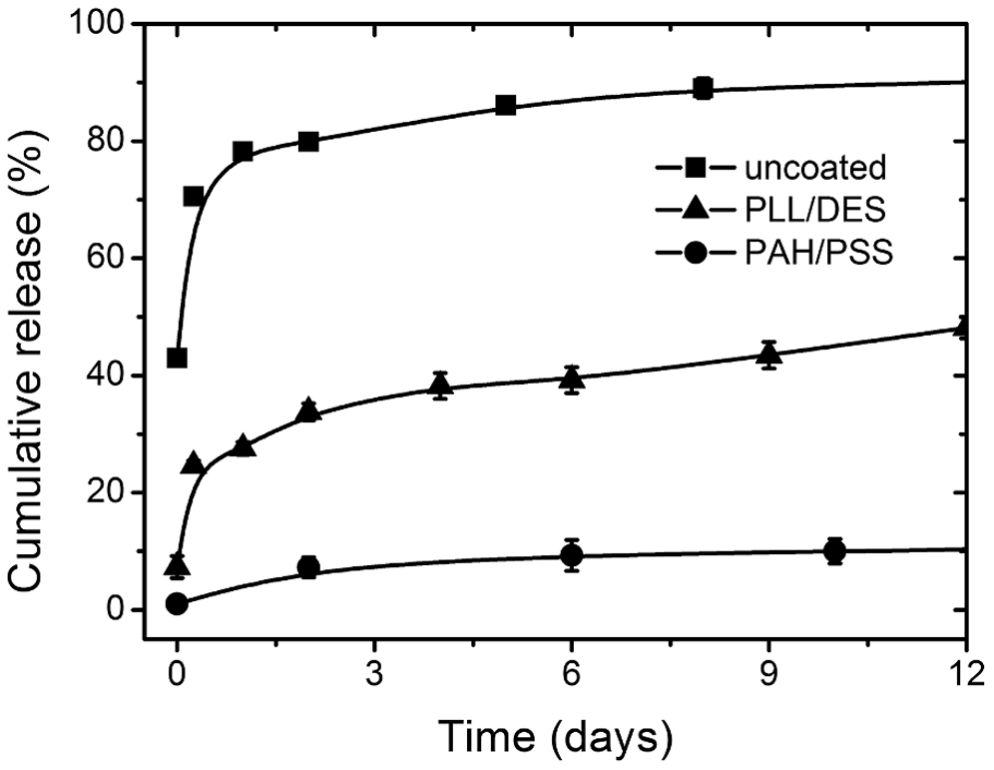
Release curve comparison between uncoated and coated nanoparticles. Coated particles were of 4 layers, with PLL/DES and PAH/PSS at 4 layers with unadjusted pH. Drug loading is 7.6±0.1%. PAH/PSS suppresses release more than PLL/DES.

**Figure 5:**
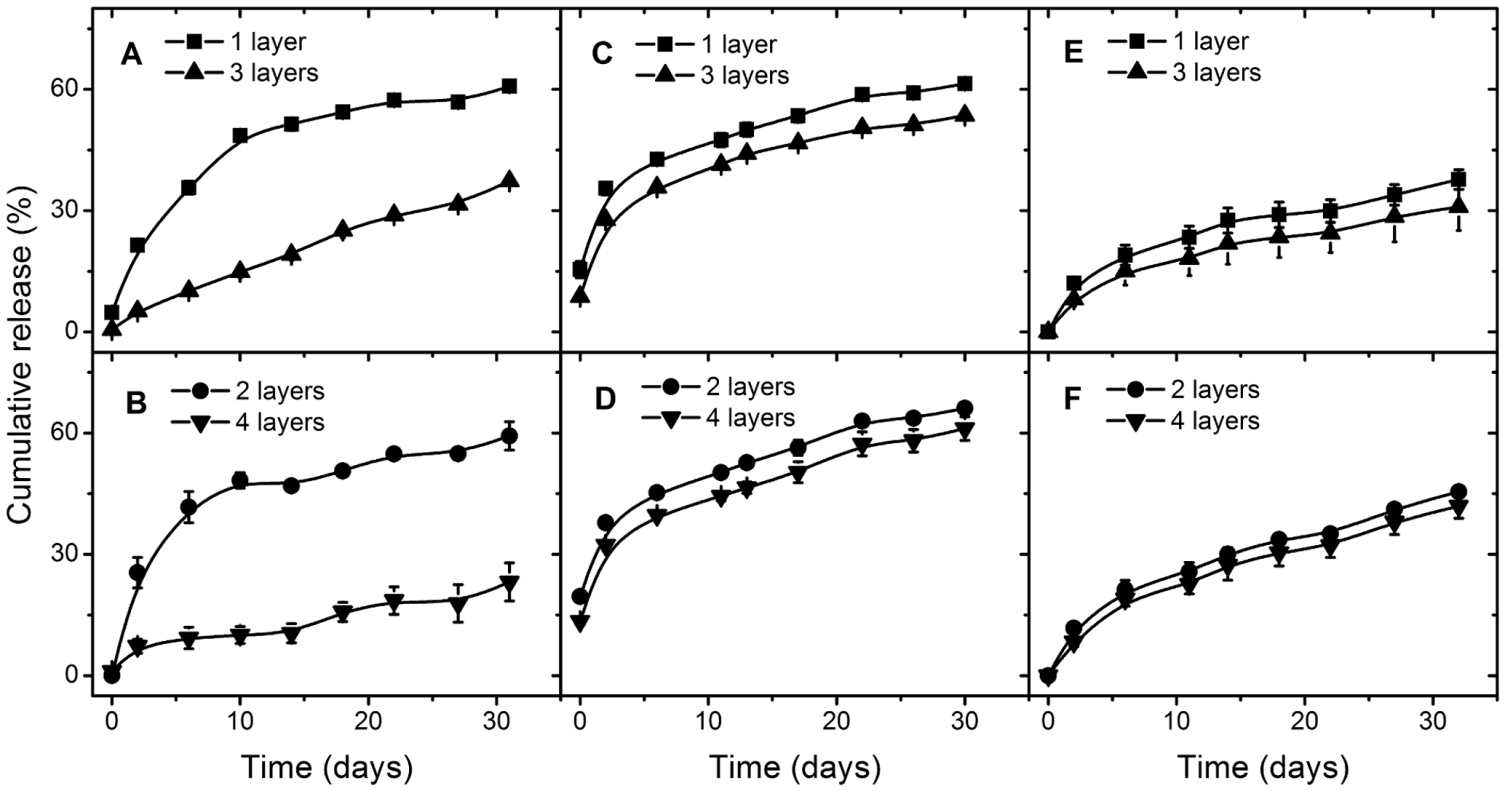
Release retardation from nanoparticles with odd and even number of layers. Release retardation from particles coated at unadjusted pH with PAH/PSS (A,B), with PLL/DES at pH 4 (C,D), and with PLL/DES at pH 9 (E,F). See Figure 4 for the release from uncoated particles. Increasing the pH during PEM build-up increases the release retardation.

PAH/PSS coating, which produces the lower amount of lost compared to PLL/DES, has higher reduction (Figure 4). Comparing the release from PLL/DES coated particles at pH 4 and 9 also shows that although pH 4 coating results in higher drug loss (Figure 3), the release reduction is actually lower than pH 9 (Figure 5), which has lower drug loss. This could be attributed to the higher permeability of the coating at build-up pH 4. It is conclusive however, that the reduction is not caused by drug loss, since it is supported by the fact that the reduction in burst release at time zero is ∼35% in absolute amount for PLL/DES, and almost completely for PAH/PSS, compared to the amount of drug loss at a mere 8%. Such loss, therefore, could not explain away the reduction on its own.

### Drug release from coated particles

Figure 4 shows the release curves of both the uncoated and coated particles with PAH/PSS and PLL/DES at unadjusted pH. When uncoated, the initial release when immediately dispersed in PBS was already 40%, and increased sharply with only one hour of incubation at 37°C. This indicates that most BSA is located on the surface, or very near to the surface, but still bound strongly enough that repeated washing with the control sample could not remove significant amount of BSA.

From the same figure, it is apparent that the release reduction afforded by PLL/DES coating is surpassed by PAH/PSS. Initial time points show that PLL/DES releases ∼40% compared to its bare particles. Both are coated without adjusted pH at 4 layers. In contrast, PAH/PSS hardly releases any. Such a marked difference cannot simply be attributed to the amount of BSA lost during the coating process, because PLL/DES showed greater loss during coating. The amount of aggregation also could not explain the reason for such markedly reduced release, since both PLL/DES coating at pH 9 and PAH/PSS coating produced similarly aggregated state but with PAH/PSS having a much higher release retardation.

For more details on each layer, the accompanying reduction of release up to 4 layers is presented in Figure 5A for PAH/PSS with unadjusted pH. The release is reduced through addition of PAH layer, while not as much through the addition of PSS layer, as seen by the relative reduction by the 2nd and 4^th^ layer from the 1^st^ and the 3^rd^, respectively. This pattern is repeated again with PLL/DES coating. Again, aggregation alone could not explain such behavior, since there is no noticeable difference in aggregation state between the two layers.

In order to investigate the effect of assembly pH on the release retardation, PLL/DES was used, since it creates less aggregation than PAH/PSS at pH 9. Note that in all instances, the release profile of the uncoated particles is much higher (Figure 4).

The release is noticeably lower in particles coated at pH 9. There are two possible factors affecting this: aggregation and the PEM layer. The higher level of aggregation in particles coated at pH 9 might contribute to the higher reduced release than at pH 4. The alternative explanation is retarded due to the increased thickness of the PEM made near the p*K_a_* of the polyelectrolyte. With less charge, more polyelectrolyte is needed in order to invert the charge of the previously built layer, resulting in higher thickness. The 2 effects might also concurrently contribute to the reduced release compared to particles coated at pH 4.

Although it is not necessarily true that higher number of layers produces lower release profile, e.g., from 1–2 layers, the increase is either lower when the layer is terminated by the same polyelectrolyte, *i.e*., 1–3, 2–4, or not significantly different. This is so regardless of the aggregation level. For instance, at build-up pH 4 where the aggregation is greater at layer 3, compared to the release profile at build-up pH 9 where the aggregation is similar for both layer 3 and 4. Regardless, layers terminated by the same polyelectrolyte yields lower release with higher layer number, or without any significant difference. In other words, adding only one layer might not result in a reduced release, but adding 2 layers does make a difference.

That a single additional layer might not significantly reduce the release, is also found in PAH/PSS coating (Figure 5A), where the difference in release retardation is with addition of 2 layers, as well as with liposomes, where those coated with (ALG/CHI)3 releases higher amount of BSA than with ALG alone [10]. The mechanism for this thus far is unknown, although this in effect means that an increase in layer number does not necessarily increase the retardation.

If the release is limited by the partitioning of the film, it means that significant difference in the partitioning is achieved only with a difference in one bi-layer. The consistent result of reducing release at any build-up pH with one bi-layer demonstrates this.

The difference in behavior of release retardation of PAH/PSS and PLL/DES might be attributable to the hydration of the PEM. It is known that polysaccharide-based PEM is highly hydrated [38]. It has also been reported that polyelectrolytes, though soluble on its own, could act as hydrophobic substance once assembled, demonstrated by preferential partitioning of curcumin into the film [39]. Due to the higher hydration of PLL/DES, PLL/DES could be more hydrophilic and the partitioning of BSA into the layer higher than PAH/PSS. BSA, therefore, more easily partitions into the PEM coating than PAH/PSS.

### Size dependence of release retardation

In order to determine whether release rate of encapsulated drug has a dependence on particle size, we attempted to use an analytical model assuming only the diffusional process without any other release-controlling mechanism. Release retardation here is defined as the ratio of release between uncoated and coated particles. To say that dependence exists implies that this ratio changes with size. Based on diffusional path lengths, smaller particles should exhibit faster release. When coated, the release could be reduced more or less than when the particle is larger. The reduction is therefore a relative value. The release from coated particles could be high, for instance, but since the release from its uncoated counterpart is similarly high, the release retardation is actually low. The opposite also holds, *i.e.* when the release is low but the retardation is high.

This discussion applies, therefore, to particles without aggregation. Intuitively, however, it is clear that additional aggregates affect release in 2 ways, by increasing the effective particle size, and by introducing additional barriers in the form of ‘shells’ in the aggregate. Release retardation therefore, is higher in aggregates compared to free-standing particles. What is attempted here, however, is to address the question of whether an increase in particle size alone could result in a reduction in release retardation.

**Figure 6:**
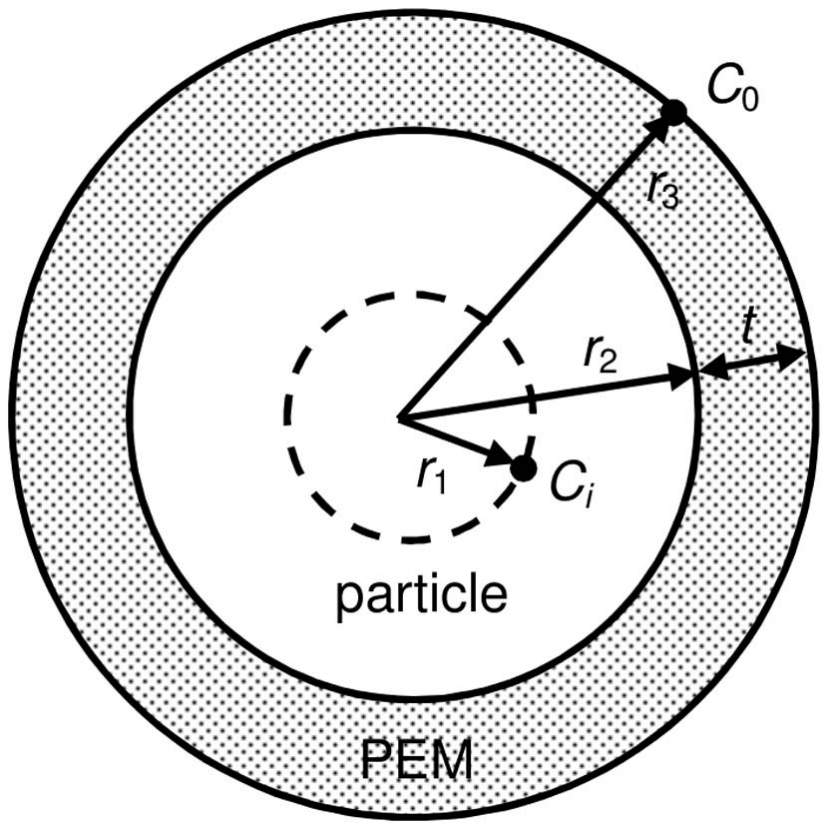
Schematic of a PEM-coated particle.

For diffusional release from composite geometry, an analytical solution for the flux on steady state has been worked out [40]. What has not been calculated, however, is the ratio between mass transfer rate between coated and uncoated particles, which we are undertaking here. The schematic for the problem is shown in Figure 6.

Although the problem is set in the context of heat transfer, it is analogous to the problem in mass diffusion, since both share the same form of elliptical equation. The assumption is simply that the mass transfer rate is at steady state, without any accumulation of drug in the PEM. The steady state assumption implies that drug is diffusing into the PEM at the same rate as efflux from the PEM. For a composite sphere, this mass transfer rate is
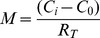



where *C* is the drug concentration. The resistance *R_T_* is the sum of individual resistance R; for each layer,
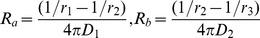



and so on for additional layers [40]. The region from an arbitrarily chosen point *r_1_* in the uncoated particle to the surface of the particle is one resistance *R_a_*, and the region from the surface of the particle to the surface of the PEM coating is another resistance *R_b_*. In total there are 2 resistances; the total resistance is
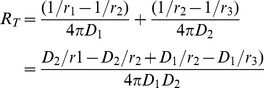



Substituting to the expression for mass transfer rate, the mass transfer rate for coated particle then is
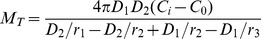



Assuming initial time points where the difference of concentration between coated and uncoated particles at *r_1_* and *r_3_* is negligible, so that the difference in concentration in uncoated particle is also *C_i_ − C_0_*, the flux for uncoated particle is simply
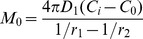



The ratio between the mass transfer rate of coated (*M_T_*) and uncoated particle (*M_0_*) becomes, with *t* being the thickness of PEM.
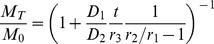



This is the ratio of the mass transfer rate at a certain time point between *r_1_* and the surface of the particle, and is descriptive only during the initial time points when *C_i_* − *C_0_* is the same for both bare and coated particles. At later time points the ratio is underestimated by the equation, *i.e.*, the real ratio is higher than prescribed, since *C_i_* − *C_0_* will become higher in coated particles than uncoated particles, due to slower release.

With 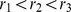, this expression has a maximum of 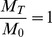. At *D_1_*  =  0, there is no diffusional resistance in the core and it becomes a capsule; and so the ratio is 1, denoting no difference in release. At *t*  =  0, coating does not exist and the ratio is also 1. When the particle size increases, *i.e.*, 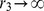, 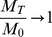, and conversely, as 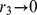, 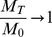.

In order to confirm the size dependence of the release retardation, particles of 2, 60, and 160 μm were prepared encapsulating BSA. The size range was increased compared to the nanoparticles in order to remove the effect of aggregation, therefore highlighting solely the effect of size on the release retardation in this case.

**Figure 7:**
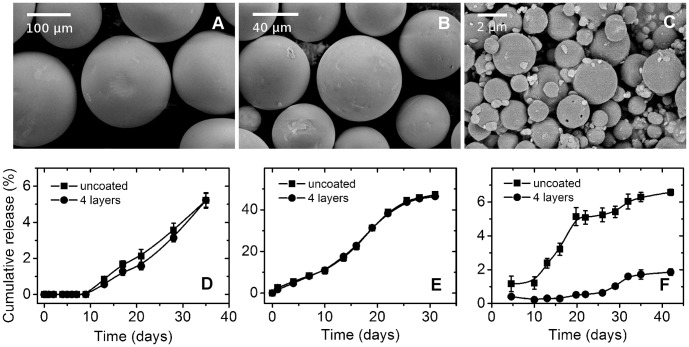
Effect of particle size on release retardation. The morphology of particles of size 160 μm (A), 60 μm (B), and 2 μm (C), is shown above their respective release profiles (D, E, F). The loading values, are, respectively, 2.2±0.2%, 5.0±0.1%, 2.6±0.1%. Release retardation is observed only at 2 μm.

The morphology of each particle set is laid out in Figures 7A–7C. The particles exhibit smooth surface characteristics with few to no pores on the surface, which have been reported in some BSA-encapsulating particles [41]. The morphology after coating remains the same for all particles.

The release from particles, shown in Figures 7D–7F, shows that coating does not affect the release for particles of size 60 and 160 μm, with non-existent BSA lost during coating. As a note, burst is also non-existent on both particles even when the drug loading is compared to FITC-dextran encapsulation, and not surprisingly, the release is slower and more sustained compared to the porous particles. This is more prominent on particles of 160 μm, which shows an extended induction time of at least 10 days, due to its larger size and lower loading compared to 60 μm.

In a simple diffusional release, smaller particle size typically results in faster release due to shorter diffusional pathway. Because of the lower loading however, the released amount is also lower _ENREF_2[42]. Release was also done in pH 9.6 buffer for other particles, but there is no significant difference between bare, coated particles, and control sample for particles of 60 and 160 μm sizes.

We also note that for microparticles ≥ 60 μm during the release studies, no particle fusion was found in coated particles. Fusion occurred in otherwise uncoated particles. That the coated particles do not experience this, then, is caused by the PEM barrier that prevents the polymer chain from being in contact with other particles. This phenomenon persists throughout all of the experiments, and indicates the persistence of PEM layer throughout.

Release reduction is only found at lower size of 2 μm (Figure 7F). The control (uncoated) sample shows higher release than the coated particle, and upon replacement of medium buffer into a higher pH at 9.6, BSA release increases approaching the level of uncoated particles, indicating that at higher pH BSA becomes more permeable. This is the first indication that release retardation works at smaller particle size.

Comparison can be made against reported works on PEM capsules that encapsulate BSA. These sometimes employ the commonly studied PAH/PSS [43] or PAH/PMA [44], but also natural polyelectrolytes such as PLL/chondroitin sulfate [45] chitosan/dextran sulfate [46], [47], or a mix of synthetic/natural polyelectrolytes such as carboxymethylcellulose/PAH [48], with layer number typically at most 10. A common kinetic feature of these capsules is that they release the load within hours (*i.e*. 5 h [44], [46], [47], 7 h [48]), initiated with a burst followed by a very slow sustained release afterwards. The same is observed in PLL/chondroitin sulfate capsule, with a burst at 30–60 min, followed by tapered release [45].

In a reported work using PAH/PSS capsules, the closest to our system, for example, the burst is 35% at 2 h,, followed by an almost flat release curve subsequently [43]. This is also similar to another PAH/PSS giant capsules (in the order of mm), with a burst of near 80% in 3 h [49], followed by what seems like an incomplete release.

This release behavior is different from capsules containing small molecular weight drugs. In those capsules, release follows a single phase with a linear increase at the beginning without any burst nor incomplete release. The main cause seems to be that with small drugs, capsules are ‘loaded’ by coating the PEM on the drug crystal as a template, as opposed to these BSA-containing capsules, which undergo cyclical pH change to open, entrap BSA from the solution, and close at higher pH. It is probable that more drugs are located in the walls of the capsules (in the case of BSA) than in the bulk, causing the burst; whereas the sustained release comes from the BSA within. Incomplete release could then be caused by BSA interaction with the matrix polymer.

Regardless, the short time of release from these capsules suggests that the permeability of PAH/PSS capsules is not as small as to act as an additional barrier for BSA release from the PLGA particles. Since permeability is, in capsules, directly proportional to the partitioning coefficient and diffusion coefficient, it could be caused then by either a relatively high diffusion coefficient in the PEM or a relatively high partitioning coefficient. Due to the low thickness of the PEM, partitioning coefficient might be the dominant factor.

In this light, the data on the micron-sized particles becomes more understandable. In the modelling approach, for bigger particles, the “resistance to permeability” within the particle is much higher than the resistance from the comparatively thinner PEM layers. Hence there is no difference in release between coated and uncoated particles. For the smaller (2 microns and below), the “resistances” becomes comparable (for the core particle and the coating), hence there is an effect due to the coating. This resistance to permeability, in our particles, comes mostly from partitioning coefficients being much less than unity.

The difference between the nanoparticle and the 2-μm particle lies mostly in the extent of burst release, which is caused by surface segregation of the drug. In the case of nanoparticles, which have a larger surface to volume ratio, this burst is increased.

## Conclusion

Release from nanoparticles is retarded in the presence of PEM coating. Aggregation exists in some samples, and the release is reduced further in such state. Comparison with particles without aggregation and those of similar aggregation but with different polyelectrolyte pair, however, shows that retardation could not simply be caused by aggregation.

With closer inspection of the analytical expression of diffusion in composite geometry, we elucidate the possibility that the effect of particle size plays a role in determining the overall diffusional resistance of the PEM. Size variation of the PLGA particles, confirms that the release retardation is diminished as particle size increases, showing size dependence of the release retardation.

We have, therefore, demonstrated that the same principle in PEM build-up on typical substrates such as silicon and glass could be applied on PEM build-up on PLGA nanoparticles with pH change and polyelectrolyte pair difference. Due to more prominent effect of release retardation with size, nanoparticles that employ PEM as a cell-targeting-moiety-anchoring platform would have a slower cargo release with respect to that without PEM, compared to similar particles of larger size.
